# Grover’s-like drug eruption in a patient with metastatic melanoma under ipilimumab therapy

**DOI:** 10.1186/s40425-016-0151-z

**Published:** 2016-08-16

**Authors:** Viktor H. Koelzer, Tobias Buser, Niels Willi, Sacha I. Rothschild, Andreas Wicki, Peter Schiller, Gieri Cathomas, Alfred Zippelius, Kirsten D. Mertz

**Affiliations:** 1 Cantonal Hospital Baselland, Institute of Pathology, Mühlemattstrasse 11, CH-4410 Liestal, Switzerland; 2 Translational Research Unit (TRU), Institute of Pathology, University of Bern, Murtenstrasse 31, CH-3010 Bern, Switzerland; 3 Division of Medical Oncology, University Hospital Basel, Petersgraben 4, CH-4031 Basel, Switzerland; 4 Cantonal Hospital Baselland, Mühlemattstrasse 11, CH-4410 Liestal, Switzerland

**Keywords:** Melanoma, Immunotherapy, Immune checkpoint inhibitors, Autoimmunity, Ipilimumab, Grover’s disease, Transient acantholytic dermatosis, Drug eruption

## Abstract

**Background:**

Dermatologic toxicity is an important adverse effect of immune checkpoint inhibitors targeting cytotoxic T-lymphocyte-associated antigen 4 (CTLA-4) and programmed cell death 1 receptor (PD-1) or PD ligand 1 (PD-L1). Skin toxicity most commonly includes a maculopapular erythematous rash and pruritus. Rarely life threatening complications such as Steven’s Johnson syndrome or toxic epidermal necrolysis may occur.

**Case presentation:**

Here we report the uncommon event of a drug-induced transient acantholytic dermatosis (Grover’s disease) in a 73-year-old Caucasian male treated with ipilimumab for metastatic melanoma. Five weeks after initiation of therapy, the patient developed a widespread polymorphic papulovesicular dermatosis on the trunk and proximal extremities with intense pruritus. Skin biopsy showed acantholytic dyskeratosis with interface dermatitis consistent with a Grover’s-like drug eruption.

**Conclusions:**

These findings should raise awareness for uncommon immune-related dermatological toxicities of immunomodulatory antibodies targeting the CTLA-4 signaling axis. We recommend biopsies of unexpected skin lesions to rapidly identify dermatological adverse events of immune checkpoint inhibitors.

## Background

Recent years have witnessed a breakthrough in the therapy of advanced melanoma. Ipilimumab, a fully humanized monoclonal IgG1 antibody targeting the immunological checkpoint surface molecule cytotoxic T-lymphocyte-associated antigen 4 (CTLA-4), has been shown to improve the overall survival of patients with metastatic melanoma in clinical trials [1–3]. Dermatologic toxicity is a common drug-related adverse event associated with this treatment. Approximately half of the patients treated with ipilimumab will experience rash and/or pruritus [4]. For most patients, dermatologic toxicity is the earliest detectable immune-related adverse event, with average onset at 3.6 weeks after the initiation of immunotherapy [5, 6]. Typical macroscopic findings include polymorphic, reticular, maculopapular, faintly erythematous rashes on the trunk or extremities and vitiligo [7]. Histologically, superficial and deep perivascular lymphocytic infiltrates consisting of CD4+ and CD8+ effector T-cells with a concomitant infiltrate of CD4+ Foxp3+ regulatory T-cells have been observed indicating a partial breach of tolerance to normal skin [8].

Grover’s disease, also known as transient acantholytic dermatosis, generally occurs among older white males (male-to-female ratio 2.4:1, mean age at diagnosis 61 years) with an incidence of 0.8 % in the hospital setting [9]. While generally accepted to be a benign, self-limited disorder, it can be persistent and difficult to manage; hence, the description of transient is misleading [9]. It usually occurs as a pruritic, polymorphic papulovesicular rash of the upper trunk and proximal extremities. Histologically, small, circumscribed foci of suprabasal acantholysis are found. The presence of scattered dyskeratotic cells, spongiosis and the level of acantholysis has been used to differentiate a Darier-like, spongiotic, Hailey-Hailey-like, Pemphigus-foliaceus-like, Pemphigus-vulgaris-like and mixed pattern [9]. An accompanying lymphohistiocytic interface dermatitis with perivascular infiltrates is common [9]. A clinically significant association between Grover’s disease and cancer, including acute leukemia, has been discovered [10, 11]. The pruritus and papulovesicular rash can be exacerbated by exercise, heat, sweating and ultraviolet light exposure. Although most studies report no association with specific drugs, individual authors have implicated interleukin 4 and D-penicillamine as disease triggers [12, 13]. However, although recognized as a common condition, the pathogenesis of Grover’s disease still remains unknown. Since it is frequently associated with other neoplastic and non-neoplastic conditions, its occurrence can be an early indicator of an underlying disease.

## Case presentation

A 73-year-old Caucasian male was evaluated for a transient ischemic attack (TIA) in May 2015. Computed tomography (CT) imaging incidentally showed multiple poorly circumscribed pulmonary nodules with a maximum diameter of 1.7 cm restricted to the upper lobe of the right lung, suggestive of malignancy. A positron emission tomography–computed tomography (PET/CT) showed intense uptake of 2-deoxy-2-(18 F)fluoro-D-glucose (18 F-FDG) in the pulmonary nodules. A diagnostic thoracoscopy with wedge resection of one nodule was carried out. Frozen section revealed a poorly differentiated neoplasia with epithelioid and spindle cell morphology of unclear etiology. Based on the differential diagnosis of a primary tumor of the lung, a lobectomy of the right upper lobe and mediastinal lymphadenectomy was performed.

**Fig. 1:**
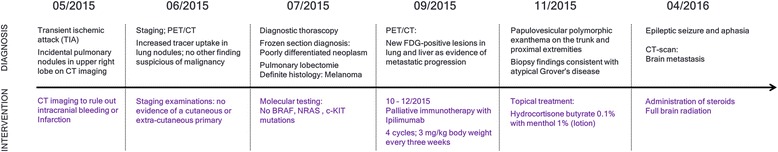
Time axis. Line graph illustrating disease progression and therapeutic intervention from primary diagnosis in May 2015 to April 2016

The formalin fixed and paraffin embedded material showed focal areas of pigment accumulation, and the tumor cells stained positive for S100, melan-A and HMB-45, consistent with malignant melanoma. Molecular testing revealed no BRAF, NRAS or c-KIT mutations. A dermatological consultation did not show an indication of a primary cutaneous melanoma. The patient was diagnosed with stage IV melanoma of unknown primary. Subsequent magnetic resonance and PET/CT imaging 2 months after lobectomy revealed a hepatic metastasis in segment II and progression of the pulmonary lesions. The patient was treated with four cycles of ipilimumab (Yervoy®), administered at a dose of 3 mg/kg body weight every 3 weeks (Fig. 1).

**Fig. 2:**
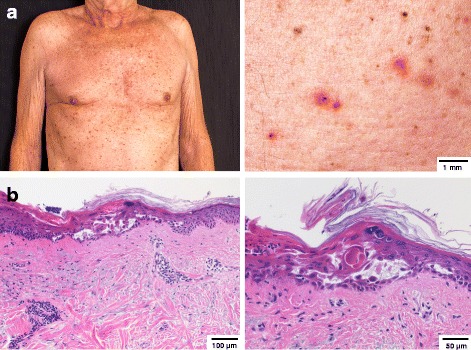
Skin findings after the second course of ipilimumab treatment. **a**) Clinical presentation. Overview image showing the diffuse eruption of a papulovesicular rash on the patient’s trunk (*left*). Detail image showing lesions of different stages ranging from early erythematous papules to crusty and scaly erosions (*right*). **b** Histopathological findings on skin biopsy (Hematoxylin-eosin stain). Overview image demonstrating circumscript acantholytic dyskeratosis and mild interface dermatitis with perivascular accentuation as an accompanying feature (*left*). Detail image showing Darier-like features with acatholytic and dyskeratotic keratinocytes present at all levels of the epidermis (*right*). Scale bars are indicated.

Three weeks after the second infusion of ipilimumab, the patient presented with a sudden eruption of a papulovesicular polymorphic exanthema on the trunk and proximal extremities, associated with a generalized and severe pruritus. On physical examination, he had multiple erythematous, 2- to 7-mm scaly papules and fragile vesicles on his chest, abdomen, back, shoulders and proximal limbs, which quickly formed crusts and keratotic erosions (Fig. 2a). A skin biopsy was performed. The predominant histological pattern was suprabasal acantholysis of keratinocytes with scattered dyskeratotic cells at various levels of the epidermis, suggestive of a Darier-like form of Grover’s disease. In addition, histological examination of the skin biopsy revealed a mild interface dermatitis and perivascular, lymphohistiocytic infiltrates with scattered eosinophils in the superficial dermis (Fig. 2b). Immunohistochemically, the infiltrate was composed of CD4+ and CD8+ T-cells with frequent but weak PD-1 expression and intermingled CD68+ macrophages (data not shown). No epidermal infiltrates were noted. Direct immunofluorescence studies for immune complex deposition were negative.

The pruritus and the rash were assessed as grade 2 toxicity, and the treatment with ipilimumab was continued according to the schedule. Of note, the patient had no recent medical history of fever, infectious conditions or abundant sweating. He also had no history of autoimmune diseases or a previous episode of Grover’s disease. The acantholytic rash and the itching were responsive to topical treatment with hydrocortisone butyrate 0.1 % lotion with menthol 1 %. However, the skin lesions persisted for more than 2 months after the end of the ipilimumab treatment. No other iplilimumab-related adverse events were noted in this patient.

After 4 cycles of treatment of ipilimumab the patient showed stable disease (RECIST 1.1 [14]) based on CT examination of the thorax and abdomen, and symptomatically experienced relief of his disease-related chronic cough, dyspnea and fatigue. In March 2016, 4 months after termination of therapy, the patient presented with stable disease on CT-scan and all skin lesions had healed. At the end of April 2016, there was appearance of new brain metastasis with epileptic seizures and aphasia. Treatment with full brain radiation and steroid infusions was initiated. There was no re-appearance of the skin lesions.

## Discussion

The modulation of signaling via coinhibitory or costimulatory receptors expressed on T-cells is a potent way to amplify antitumor immune responses. This approach has been exploited successfully for the generation of monoclonal antibodies against the immune checkpoints CTLA-4 and PD-1/PD-L1. Because checkpoint blockade does not just enhance tumor-specific immune responses, autoimmunity can occur as an adverse event. Inhibition of the PD-1 and CTLA-4 feedback loop commonly induces a dysregulation of the immune system associated with severe and even fatal immune-related adverse events due to T-cell activation and proliferation. While any organ system may be involved, cutaneous side effects are frequent. Here we report the case of a 73-year-old male patient with metastatic melanoma treated with ipilimumab who developed a widespread pruritic eruption with histopathologic findings of acantholytic dyskeratosis, consistent with a Grover’s-like drug eruption.

Grover’s-like drug eruptions have been previously associated with the use of BRAF inhibitors such as vemurafenib [15] and dabrafenib [16]. The etiology of this finding is unknown, but given its relative frequency in patients treated with BRAF inhibitors, paradoxical activation of the MAPK pathway might play a role. Most of the patients who developed drug-induced cutaneous reactions under BRAF inhibitors responded to topical emollients or corticosteroids, with only a few severe cases requiring oral corticosteroids or retinoids [17].

A Grover’s-like drug eruption under immune-checkpoint inhibitor treatment has been previously reported in only one single patient who received ipilimumab for stage IV melanoma [18]. The here presented case differs from this previous report and extends the characterization of ipilimumab-induced acantholytic dermatosis in several aspects: First, no characterization of cutaneous immune infiltrates in ipilimumab-induced Grover’s disease was previously attempted. Here we show a co-existence of typical features of acantholytic dermatosis with low-grade interface dermatitis and perivascular lymphocytic infiltrates composed primarily of CD4+ and CD8+ T-cells. Increased dermal and perivascular lymphocytic infiltrates have been previously reported as a feature of ipilimumab-induced rash and may indicate partial breach of tolerance to normal skin [8]. Increased superficial perivascular infiltrates are also frequently identified in Grover’s disease [9]. Taken together, these coincidental findings may indicate a potential pathogenic involvement of the T-cell response in the development of Grover’s disease and merit further investigation.

Second, the exanthema in our patient was very extensive, affecting the patient’s entire trunk and proximal extremities. The size of the skin lesions varied considerably, and the rash and pruritus of the patient did not resolve immediately and completely after ipilimumab disruption, but only with a delay of 2 months. Our case is similar to the previously reported case with respect to the absence of other medications and other causes for abundant sweating, which could represent other causative triggers for the patient’s skin manifestation. Similarities are also found in an association of the Grover’s-like drug eruption with therapeutic response to immune-checkpoint inhibitor therapy. Further analyses are required to clarify whether drug-induced Grover’s disease may represent a form of on-target dermatologic toxicity that is linked to clinical efficacy.

## Conclusions

Patients treated with immune checkpoint inhibitors – the CTLA-4 antibody ipilimumab or anti-PD1/-PD-L1 inhibitors – require continuous dermatologic monitoring throughout their treatment. A timely classification of dermatologic toxicity into harmless or life-threatening events is needed to consider interruption of immunotherapy and introduction of immunosuppressive drugs. Ipilimumab-induced Grover’s disease is a rare complication, histologically characterized by acantholytic dyskeratosis with mild interface dermatitis and perivascular lymphocytic infiltration. Based on the presently available evidence, this immune-related adverse event follows a benign course, responds well to symptomatic treatment and does not require interruption of immune checkpoint inhibitor therapy.

## Abbreviations

18 F-FDG, 2-deoxy-2-(18 F)fluoro-D-glucose; CD, cluster of differentiation; CT, Computed tomography (CT); CTLA-4, cytotoxic T-lymphocyte-associated protein 4; PET/CT, positron emission tomography–computed tomography (PET/CT); PD-1, programmed cell death protein 1.
